# EEG theta responses induced by emoji semantic violations

**DOI:** 10.1038/s41598-021-89528-2

**Published:** 2021-05-12

**Authors:** Mengmeng Tang, Xiufeng Zhao, Bingfei Chen, Lun Zhao

**Affiliations:** 1 School of Foreign Languages, China University of Petroleum, Beijing, China; 2 School of Educational Sciences, Liaocheng University, Liaocheng, China

**Keywords:** Neuroscience, Psychology

## Abstract

This study investigated emoji semantic processing by measuring changes in event-related electroencephalogram (EEG) power. The last segment of experimental sentences was designed as either words or emojis consistent or inconsistent with the sentential context. The results showed that incongruent emojis led to a conspicuous increase of theta power (4–7 Hz), while incongruent words induced a decrease. Furthermore, the theta power increase was observed at midfrontal, occipital and bilateral temporal lobes with emojis. This suggests a higher working memory load for monitoring errors, difficulty of form recognition and concept retrieval in emoji semantic processing. It implies different neuro-cognitive processes involved in the semantic processing of emojis and words.

## Introduction

Human communication is based on a complex expressive system and normally occurs embedded within an interactional exchange of multi-modal signals^1,2^, including both language (phonemes, words, phrases, sentences, etc.), and paralanguage (various visual and vocal modalities). Among all the paralinguistic cues that have been studied so far, emojis, which refer to picture characters or pictographs, have witnessed a sharp increase in computer-mediated communication contexts. According to a 2015 study by the British app developer, SwiftKey, which collected and examined over a billion bits of data from Android and iOS devices in sixteen languages, 45 percent of all messages contained all kinds of emojis^3^. Emojis allow one to deliver nuances of meaning in more compact and holistic ways, so it is a compressed, economical system for rapid communication. And this visually based version differs from pictures or other symbols as it has commonly-accepted intrinsic meanings, can be used adjunctively or substitutively within a written text, and is able to be combined in larger structures. At the same time, they differ from linguistic information in that they are not within the scope of phonetics, morphology, or lexical analysis. These special features make the research on emojis of great significance from the perspective of revealing cognitive processes in human’s multi-modal communication.

However, despite its significance and unique features, previous literature mainly centered on language processing, like morphology recognition, lexical retrieval, semantic integration and etc., and few accounts on the neuro mechanisms of paralanguage semantic processing. It has been hypothesized from the socio-cultural level that in communications with emojis, these visual tokens may not require a large cognitive effort to retrieve and select words, phrases, or use world knowledge associated with the discourse practices^3^, but no electrophysiological evidence was provided. The EEG oscillations in specific frequency bands induced by cognitive events is believed to reflect the dynamic recruitment of related neuronal networks involved in cognitive processing^4^. EEG oscillations induced by semantic congruency of emojis within a sentential context can expose in-depth information of the cognitive demands in several stages of emoji semantic processing: form recognition; lexical retrieval and selection; and contextual integration. By computing the discrepancy between incongruent and congruent conditions, physical differences can be eliminated, which provides a possibility to compare emoji and word processing. Concerning the widely used emojis, the current study aims to explore the semantic processing of emojis in Chinese sentential context by investigating the EEG activities induced in congruent/incongruent conditions.

Even though an integrated understanding of linguistic processes based on the detailed knowledge of the corresponding neural networks is still incomplete, it has been widely reported that the theta-band oscillations (TBO) (4–7 Hz) play an important role in the activation of neuronal assemblies, representing concepts and words. Given that a robust relation was observed between theta power oscillation and working memory^5–10^, or memory encoding/retrieval^11–15^, the TBO corresponding to various linguistic manipulations implies processes at the interface between memory and language in language comprehension. For instance, retrieval of language-related visual information (e.g., word forms in long-term memory), retrieval of lexical semantic information (e.g., word meaning in long-term memory), integration of lexical meaning into context (e.g., working memory), and etc. These processes in language comprehension were reflected as TBO in topographical distribution.

The frontal midline theta increase has been proposed to relate to the integration of lexical meaning into context or an expanded explanation of error monitoring or working memory. Repeated evidences were from theta oscillation in frontal area. Semantic anomaly processing can elicit theta band power increase^16–23^. It was observed during 300–800 ms after critical word onset over midfrontal areas for the semantic violations only^18,19^, even though some are with relatively higher amplitude^17^, while some with low amplitude (2–5 Hz)^22^. But the theta power may not increase for certain semantic anomaly^24^. Unlike the above-stated studies in Dutch, semantic violations in Italian^25^ and Chinese^26^ which have more flexible word order, did not induce increased theta power. It has been argued that the language type, L1 or L2, task demand, or the method employed in time–frequency analysis may account for the inconsistent findings of theta power for semantic violation.

Moreover, thematic (e.g., leash-dog) over taxonomic (e.g., horse-dog) relationships^27^, both open class (OC) and closed class (CC) words^11^, led to the theta power increase. Theta power increase was also found with syntactic anomalies^21,28–32^ and cognitive control^33^. Therefore, theta power increase in frontal area may index greater processing efforts, errors, or unexpectations in types of integration, such as lexicon-context, thematic relations, and etc.

The increase in bilateral temporal area may be associated with the lexical-semantic information retrieval, and that in occipital area suggests the visual form retrieval. Bastiaansen et al.^11^ observed a theta power increase over left temporal areas for OC words, but not for CC words. It was argued to reflect the activation of a network involved in retrieving the lexical–semantic properties in temporal area because it is more difficult to retrieve the diversified usages from OC words. And the larger left-occipital theta increase with OC words was argued to reflect the longer and more complex visual forms than CC words. This was further confirmed in Bastiaansen et al.^34^, in which temporal electrodes showed larger theta power increases in processing auditory semantic properties, while occipital area showed larger theta responses with visual semantic properties.

As mentioned above, the direction of the theta oscillation (increase or decrease) associated with semantic violations is not consistent and the oscillatory neuronal dynamics in theta band is related to the functional network that serves the role of retrieving visual forms, lexical-semantic properties, and integrating different sources of information in understanding linguistic input. Emojis fall outside the boundaries of morphology and lexical analysis, and whether the semantic processing of emojis is also related to the theta power oscillation remains unclear. In this study, we will conduct the wavelet-based time–frequency analysis concerning the neural differentiation between emoji and word processing, and more specifically, the modulation of oscillatory neuronal activity and typographic distribution of theta band oscillation induced by processing congruent/incongruent emojis in Chinese sentential context, with a contrast of congruent/incongruent words. On basis of previous studies, we expect a stronger increase of theta activity^18,19^ to be associated with semantically incongruent words as compared to congruent words, and we hypothesize that the semantic processing of emojis may also be reflected in theta band power oscillation. This study will provide a window to expose how multi-modal information are processed in human brain, and what types of cognitive demands are needed in processing different models of information, and therefore contributes to the wider category of neuro mechanisms of multi-modal communication of human beings.

## Results

### Anterior–posterior regions

**Figure 1:**
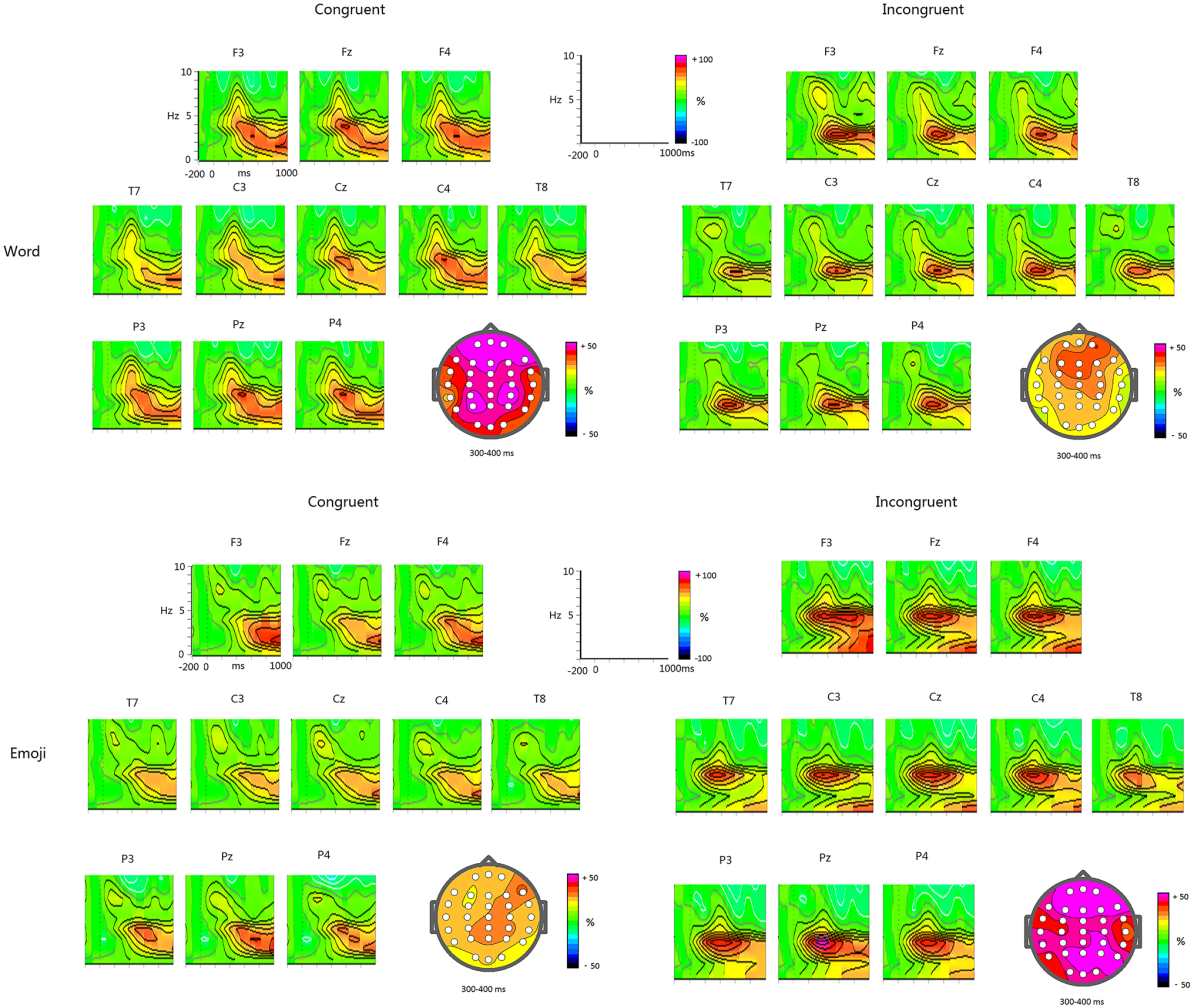
TBOs for word and emoji conditions as well as the 2D scalp mapping, respectively.

**Figure 2:**
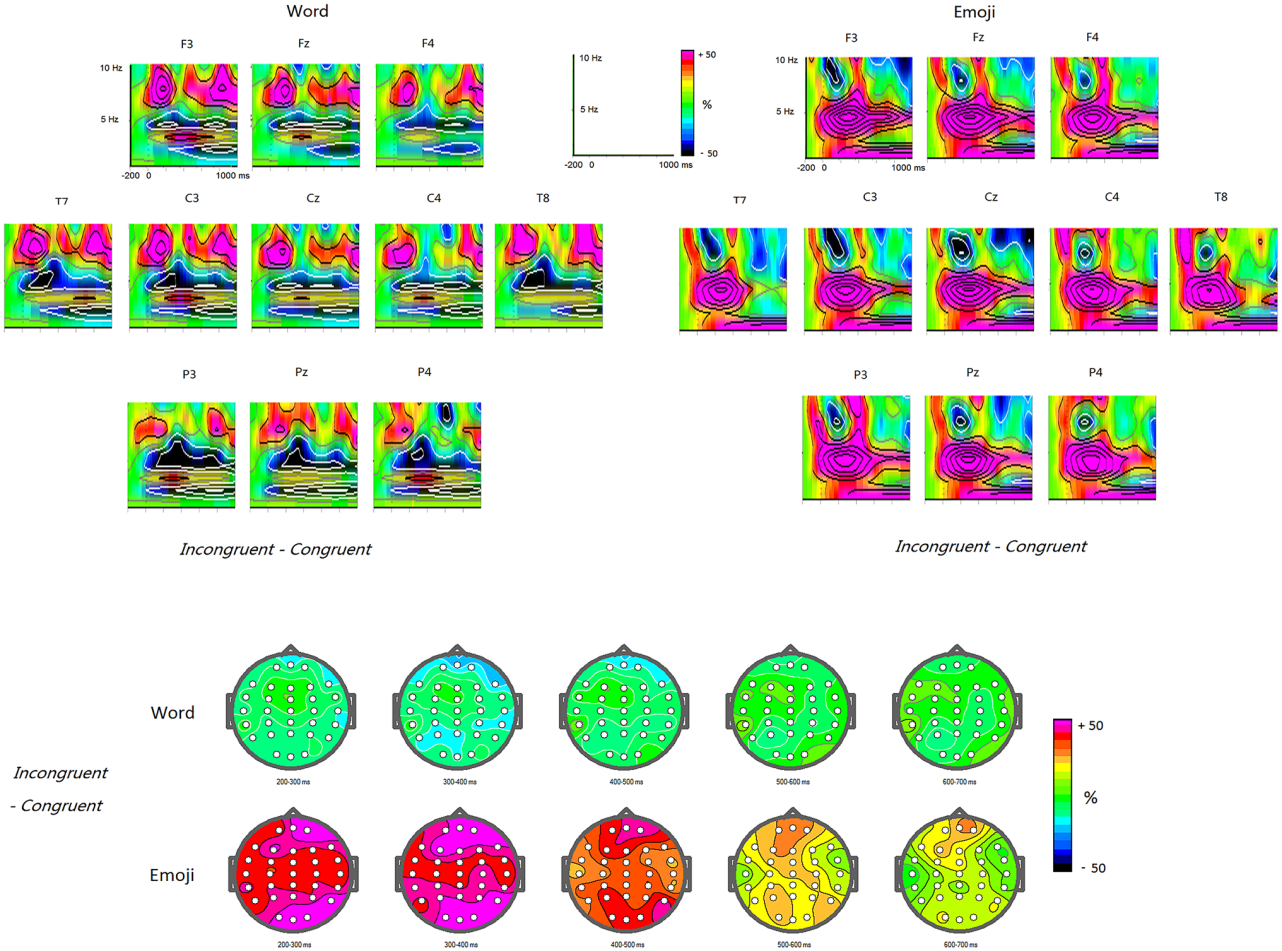
The differences of TBOs and ABOs between incongruent and congruent conditions for word and emoji conditions as well as the 2D scalp mapping, respectively.

The main effect for Category was significant, F(1,21) = 14.596, p < 0.005, Partial ƞ^2^ = 0.41, indicating the TBO higher for emoji (83.6%) than for word stimuli (64.5%; Figs. 1 and 2). This category effect was qualified by an interaction between Category with Congruency, F(1,21) = 29.88, p < 0.001, Partial ƞ^2^ = 0.587, revealing that for congruent condition, the category effect was not significant (p = 0. 615), while for incongruent condition, emoji elicited higher TBO (98.3%) than did words (59.5%; p < 0.001) and that incongruent words induced slightly lower TBO than congruent condition (70.1%; p > 0.1), whereas incongruent emojis induced higher TBO than congruent condition (67.2%; p < 0.001). In addition, both main effects of Anterior–Posterior distribution [F(2,42) = 9.16, p < 0.005, Partial ƞ^2^ = 0.304] and Laterality [F(2,42) = 9.75, p = 0.001, Partial ƞ^2^ = 0.317] were significant and particularly, the two-way interaction of Anterior–Posterior distribution by Laterality was also significant, F(4,84) = 4.77, p < 0.02, Partial ƞ^2^ = 0.185 , showing a maximum of 77.8% at Cz site. No other effects reached significant level (ps > 0.1).

### Temporal regions

Similar to anterior-parietal regions, the TBO in temporal regions was also higher for emoji stimuli (85.8%) than for word stimuli (64.4%; see Figs. 1 and 2). The statistical significance of this pattern was determined by a main effect for Category [F(1,21) = 24.06, p < 0.001, Partial ƞ^2^ = 0.534]. Although the main effect of Congruency did not reach significant level (F < 1), the two-way interaction of Category * Congruency was significant, F(1,21) = 26.01, p < 0.001, Partial ƞ^2^ = 0.553. Further analysis for this interaction showed that for congruent condition, the category effect was not significant (F < 1), while for incongruent condition, emoji elicited higher TBO (96.6%) than did words (64.0%; p < 0.001) and that incongruent words induced lower TBO than congruent condition (74.2%; p < 0.05), whereas incongruent emojis induced higher TBO than congruent condition (72.8%; p < 0.001). No other effects reached significant level (ps > 0.1).

Moreover, as shown in Fig. 2, the alpha-band oscillations (ABO) was enhanced for incongruent-congruent words (p < 0.05) and decreased for incongruent-congruent emojis (p < 0.05); hence, there was a significant interaction of Category * Congruency (p < 0.02).

## Discussion

The present study investigated the semantic processing of emojis via the technique of wavelet-based time–frequency analysis. The last segment of experimental sentences was designed as either words or emojis functioning as adjectives for the expression of comments consistent or inconsistent with the context. The aim was to characterize the nature, i.e., magnitude and scalp topography of EEG power changes that were expected to occur as a result of semantic violation of emojis. We zoomed in on the theta band brain dynamics (4-7 Hz). The results showed different theta activities between the presentation of the incongruent emojis and the incongruent words within sentential contexts. Moreover, the congruent emoji induced the theta power increase at the mid frontal, occipital, and temporal areas, which are related to the cognitive demands. These results suggested the neural dissociation for the semantic processing between emojis and words.

One notable aspect is that like prior findings of theta power increase in frontal area with improved cognitive processing, both word and emoji processing led to an increase. Previous research has found that the theta band is associated with improved cognitive processing of linguistic information, sustained attention, increased mental effort or task difficulty, extensive use of memory mechanisms^11,17–19,27,35,36^. The current results prove that the cognitive processing of paralanguage information, which falls out of the category of phonetical, morphological, and lexical analysis is also associated with the theta power oscillation. It suggests that TBO indexes the general activation of neuronal assemblies, representing meaningful concepts. The TBO differences between emojis and words in the congruent condition may be due to the physical differences between them. That is, emojis are likely to be more unexpected in sentences, and their meanings are more ambiguous with nuances, and therefore their processing require more efforts in recognition and meaning integration, and accordingly led to higher burden of working memory.

To avoid the physical differences, our focus was on the discrepancies between incongruent minus congruent emoji processing and incongruent minus congruent word processing. Semantic violations with emojis led to a conspicuous theta power increase while incongruent words induced slightly lower TBO than congruent conditions in midline frontal areas. The degree of theta power increase with incongruent emojis shows a gradual decline from the fronto-central midline to the peripheral frontal area. It has been stated that the theta power increase in frontal area implies greater processing efforts and difficulties in integration, such as lexicon-context, thematic relations, word length-context and etc.^11,26,27^. Combining these findings with our emoji-related frontal theta effects, we argue that the current finding with emojis suggests a higher processing difficulty in integrating mismatched emojis into sentential contexts. And it led to a larger working memory load in error monitoring which elicited a dramatic and prolonged increase with the theta frequency largely spread in frontal area. In contrast, the slight decrease of TBO with incongruent words shows inconsistent results with some prior research on semantic violations^17–19,22^, but similar with Braunstein et al.^24^, Luo et al.^26^, and Penolazzi et al.’s^25^ findings. It is possibly because of the language structure, and the way to show semantic violation, which makes the less sensitivity of theta oscillation in processing Chinese incongruent words. Studies which observed the theta band effect were mainly carried out in Dutch, while Chinese has more flexible language structure, more diversified way of interpretations and different writing system. An alternative explanation is about the position of target words, or the way to form a semantic anomaly. In prior literature, such as Hald et al.^19^, the semantic anomaly is usually an unexpected adjective (e.g., sour) to the noun (e.g., train), which form word-to-word matching violations semantically. While in the present study, thematical incongruency (e.g., unhappy) was presented to the context (e.g., I successfully passed the exam) and the semantic anomaly was formed via abnormal comments. However, obviously, emoji processing was not affected by these factors, and showed a high sensitivity.

In occipital area, emojis also produced a relatively strong increase of theta power in comparison with words. Bastiaansen et al.^11^ found that the theta power in left occipital lobe is significantly stronger for processing open-class vocabulary than closed-class vocabulary. It was explained as that the left occipital area may be related to the visual form processing, because open-class vocabulary is longer and more complex. This was further confirmed in Bastiaansen et al.^34^, in which occipital area showed larger theta responses with visual semantic properties. In terms of forms, emoji does not have the normal morphology of linguistic information, but pictographic and ideographic. We hypothesize that the theta power increase with emoji processing in occipital area reflects the difficulty and complexity in emoji visual-form retrieval.

In addition, we also observed an obvious theta power increase in bilateral temporal lobe with incongruent emojis. It has been reported that theta power increase in the temporal lobe reflects lexical retrieval, associated with long-term memory^11,15^. Emojis fall outside the category of lexical analysis, but they have intrinsic meanings. In a survey of 70,000 emoji-containing tweets in 13 languages, Novak et al.^37^ found that emojis provide a wide array of semantic nuances to written texts. In another word, emojis have pre-constructed concepts that need to be retrieved in semantic processing. From the increase of theta power, we hypothesize that the concept retrieval of emojis is relatively difficult because an emoji has diversified interpretations, corresponding to more than one lexical meaning. For example, a smiling emoji may refer to the verb “smile”, the adjective “happy”, the noun “smile” and etc. So, it might be more difficult to retrieve the lexical meaning of an emoji, and that has been reflected on the intensive increase of theta power on both sides of the temporal lobe.

Although it is beyond the purpose of this study, it is also noteworthy that ABO is increased for incongruent-congruent words and decreased for incongruent-congruent emojis. ABO has been noticed in phrase structure violations^17^, semantic violations^22,26^ in language processing, and is also associated with the attention process, i.e., more attention usually triggers ABO suppression^38^. Therefore, ABO may reflect the processing of phrase structure or semantics to some extent, and it is also likely to be the reflection of higher attention triggered by the syntactic or semantic abnormalities themselves. In the current study, we tentatively hypothesize that the ABO decrease with emojis suggests the higher attention with these abnormal forms in sentences. Since this is not the focus in the current study, the role of ABO and its association with multi-modal information processing remains open for future explorations.

Since changes of induced EEG activity can be employed to investigate interactions between neuronal webs underlying linguistic processing, induced oscillatory activity provides a valuable way to explore phenomena involved in functional networks formation, activation, and uncoupling. These results suggest that the neural activities of semantic processing for emojis and words differ not only in theta power oscillation patterns but also in the neuronal network related to certain processes in comprehension. The wide topographic distribution of the theta power changes with emojis, hints at the existence of multiple neural generators including the recognition of forms, retrieval of pre-constructed concepts, and integration of emoji meaning into contexts. More specifically, the emoji recognition and concept retrieval related to long-term memory are reflected as theta power increase in the occipital and bilateral temporal lobe. The integration of emojis to context requires high working memory, shown as the theta power increase in the frontal lobe. We tentatively hypothesize that semantic processing of paralanguage information may involve similar cognitive processes as linguistic information, such as activation, integration, and selection (BIAS)^39^; however, these processes in emoji processing require more cognitive efforts in comparison with language. Therefore, contrary to the theoretical idea that emoji processing may not need to retrieve or select lexical information and is easier^3^, we provided electrophysiological evidence that its processes are not fewer but may be more complex and difficult in comparison with language processing.

To sum up, this is the first time–frequency analysis on emoji semantic processing, and theta band oscillation was found to differ from that in word processing in sentential context. This study revealed the nature of emoji semantic processing, including the magnitude and scalp topography which shows a neuronal network with different functional roles. The current research contributes to the knowledge of EEG oscillation by expanding the focus to paralanguage semantic processing and providing further evidence on the functional role of theta power. It also contributes to the wider category of cognitive demands of human communication, by exposing how multi-modal information are processed in human brain, and more specifically, what types of cognitive demands are needed in processing different modals of information. It therefore sheds some lights on the neuro mechanisms of human communication, which is one of the most difficult subjects in scientific exploration.

## Conclusion

The present study explored the neurocognitive basis of emoji semantic processing via EEG experiments. EEG analysis showed that incongruent emojis led to a higher theta power increase than congruent conditions. Furthermore, the theta power increase was observed at midfrontal, occipital and bilateral temporal electrodes with emojis. These results may suggest the cognitive processes of form recognition, concept retrieval, and meaning integration in emoji semantic processing and the higher requirement of cognitive efforts involved in these processes. Further study with other electrophysiological evidences will lead to a more precise understanding of emoji processing.

## Method

All methods were carried out in accordance with relevant guidelines and regulations. All experimental protocols were approved by the Academic Ethics Committee of China University of Petroleum (Beijing).

### Participants

Twenty-two participants (12 females; average 22.5 years old) from China University of Petroleum (Beijing) participated in the experiment. All participants were right-handed, with normal or corrected vision, and no neurological or psychiatric disorders was reported. The ethic form was in accordance with the International Ethical Guidelines for Health-related Research Involving Humans, and was approved by the Academic Ethics Committee of the university where participants took part in the experiment. All participants signed an informed consent to participate in this study.

### Stimuli and procedure

In order to eliminate the differences essential to semantic processing, the familiarity and acceptance of emoji stimuli were carefully controlled. In a pre-test, 63 Chinese participants (who did not participate in the EEG experiment) were asked to do a rating task on 20 commonly-used emojis in social networking apps, according to the degree of familiarity and acceptance to the emoji-word mapping on a scale from 0 (least acceptable/least familiar) to 9 (most acceptable/most familiar). Six emojis with highest frequency and acceptance were finally chosen and used in our EEG experiment. The rating results are presented in Table 1. The results showed that there were no significant differences in the familiarity to these emojis and the meaning of words and emojis are highly matched.

**Table 1:** Ranking results of emojis in the experiment.

Emojis	Happy (M, SD)	Sad (M, SD)	Angry (M, SD)	Glad (M, SD)	Good (M, SD)	Bad (M, SD)	Difference (ANOVA)
Familiarity	8.05(2.05)	8.03(2.04)	8.24(1.91)	8.03(1.99)	8.4(1.72)	7.57(2.45)	F (5,62) = 1.17, p = 0.32, > 0.05
Acceptance	7.63(2.31)	7.59(2.39)	8.16(2.02)	8.06(1.63)	8.24(1.72)	7.35(2.36)	F (5,62) = 1.89, p = 0.10, > 0.05

Sixty contexts for the 6 target emojis and their verbal counterparts which were two-character Chinese adjectives (see Table 2) were designed as test materials. All test sentences were divided into 8 segments, with the last segment as the critical segment. The critical word length was matched across sentences, and the congruent/incongruent sentences matched for linguistic variables. Test sentences are common in daily usage in Chinese, and the final comments are based on normal emotions to contexts. All critical words are ranked in the 8000 high-frequency words in *Modern Chinese Frequency Dictionary*^40^. In addition, Table 3 shows the frequency counts of these words in the BCC Corpus (Beijing Language & Culture University Corpus Center, http://bcc.blcu.edu.cn/) with a total of 13 billion Chinese characters. The frequency counts of congruent and incongruent words have no significant differences (t = 1.456, p = 0.219). The following are examples of the stimuli.

**Table 2:** Target emojis and their verbal counterparts in matched and unmatched conditions.

Congruent condition		Incongruent condition	
Emoji	Verbal counterpart	Emoji	Verbal counterpart
	happy (开心)		sad (伤心)
	sad (伤心)		happy (开心)
	angry (生气)		glad (高兴)
	good (很棒)		bad (很差)

**Table 3:** Frequency counts of critical words.

Critical word	Pinyin	English	Word length	Frequency in sub-corpora					Total frequency counts
				Literature	Dialogues	Multi-domain	Periodicals	Press	
高兴	gaoxing	glad	2	26,228	35,534	126,260	88,091	98,325	374,438
开心	kaixin	happy	2	3603	272,635	200,864	5256	25,171	507,529
伤心	shangxin	sad	2	5858	34,720	44,571	3274	20,611	109,034
生气	shengqi	angry	2	9993	41,072	61,581	11,318	41,053	165,017
很棒	henbang	good	2	302	28,463	12,868	251	1177	43,061
很差	hencha	bad	2	314	4045	6985	4652	4102	20,098
Total character counts in five corpora				13,000,000,000					

#### The congruent emoji condition

期末/考试/顺利/通过了, /我/感到/非常/

Final/exam/successfully/passed,/I/felt/very/

I successfully passed the final exam, and I felt very

#### The congruent verbal condition

期末/考试/顺利/通过了, /我/感到/非常/开心。

Final/exam/successfully/passed,/I/felt/very/happy.

I successfully passed the final exam, and I felt very happy.

#### The incongruent emoji condition

期末/考试/顺利/通过了, /我/感到/非常/

Final/exam/successfully/passed, I/felt/very/

I successfully passed the final exam, and I felt very.

#### The incongruent verbal condition

期末/考试/顺利/通过了, /我/感到/非常/伤心。

Final/exam/successfully/passed,/I/felt/very/sad.

I successfully passed the final exam, and I felt very sad.

In the experiment, participants were asked to read the sentences on the screen. Each trial was displayed segment by segment in the center of the monitor. For the first 7 parts each lasted for 800 ms, and the 8th segment displaying the target emoji /word lasted for 1000 ms. The interval between the two trials was 1500 ms, and a total of 240 trials were randomized under these four conditions.

### EEG recording

Electroencephalogram (EEG) signals were continuously recorded by the NeuroLab digital amplifier system, with NeuCap of Ag /AgCl electrodes at 64 sites according to the expanded international 10–20 system (Yiran Sunny Technology Co. Ltd, Beijing, China, http://www.neurolab.com.cn). The reference electrode was placed on the tip of nose. It was converted into bilateral mastoids for reference in data analysis. To record vertical and horizontal electro-oculogram (EOG) signals, two electrodes were placed above and below the right eye respectively, and two electrodes at the left and right outer canthi. The impedance of the electrodes was kept below 5kΩ. EEG and EOG signals were amplified with a band pass of 0.1–100 Hz at a sampling rate of 1000 Hz.

### Data analysis

Data were analyzed using WinEEG software (www.mitsar-eeg.com) as well as house-made Matlab routines. Raw EEG data were 1.0 Hz high-pass filtered (24 dB). Eye movements were corrected using an ICA procedure (WinEEG software; www.mitsar-eeg.com). Remaining artifacts exceeding ± 100 μV in amplitude or containing a change of over 100 μV within a period of 50 ms were rejected. The EEG was then segmented into epochs ranging from 300 ms before to 1000 ms after stimulus onset for all conditions.

TBO, i.e., event-related de-synchronization (ERD/S), was calculated by applying a wavelet analysis to individual trials and averaging the time frequency plots (WinEEG software; www.mitsar-eeg.com). This procedure was applied to frequencies ranging from 4 to 7 Hz in steps of 0.5 Hz (theta band) and the amplitudes were extracted and baseline-corrected yielding time–frequency plots.

**Figure 3:**
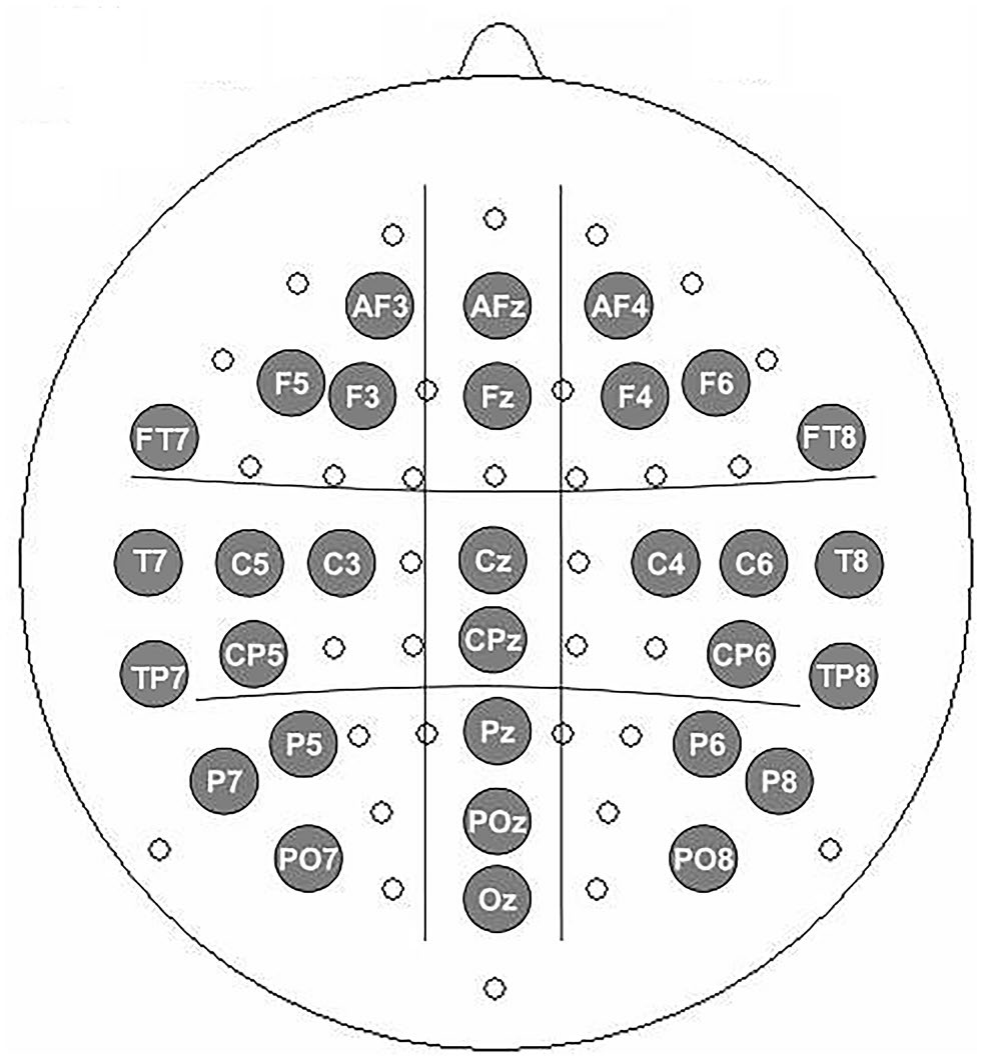
Electrode montages for the different scalp distribution of TBO computation.

The scalp distribution of this TBO was widely spread, and therefore, our main analysis compared the mean TBO in left, midline and right clusters of electrodes at anterior, center and posterior regions as well as bio-temporal regions (see Fig. 3). We calculated the mean TBO during an epoch between 200–500 ms post stimulus onset and the measurements were subjected to ANOVAs with repeated measures in order to determine statistical reliability. For the anterior–posterior regions, the factors were stimulus Category (word, emoji), Congruency (congruent, incongruent), Anterior–Posterior distribution (anterior, center, posterior), and Laterality (left, medial, right). For the temporal regions, the factors were stimulus Category (word, emoji), Congruency (congruent, incongruent), and Laterality (left and right). In all the analyses, degrees of freedom and MSEs were corrected for non-sphericity using the Greenhouse–Geisser correction (for simplicity, the uncorrected degrees of freedom are presented).
